# A *Narnavirus* in the Trypanosomatid Protist Plant Pathogen *Phytomonas serpens*

**DOI:** 10.1128/genomeA.00711-16

**Published:** 2016-07-28

**Authors:** Natalia S. Akopyants, Lon-Fye Lye, Deborah E. Dobson, Julius Lukeš, Stephen M. Beverley

**Affiliations:** a Department of Molecular Microbiology, Washington University School of Medicine, St. Louis, Missouri, USA; b Biology Centre and Faculty of Sciences, Institute of Parasitology, University of South Bohemia, České Budějovice, Czech Republic; c Canadian Institute for Advanced Research, Toronto, Ontario, Canada

## Abstract

We describe here a new RNA virus (PserNV1) from the plant protist parasite *Phytomonas serpens* (family *Trypanosomatidae*, *Kinetoplastida*, supergroup *Excavata*). The properties of PserNV1 permit assignment to the genus *Narnavirus* (*Narnaviridae*), the first reported from a host other than fungi or oomycetes.

## GENOME ANNOUNCEMENT

Viruses within the family *Narnaviridae* are composed of a positive single-stranded RNA segment (2.3 to 3.6 kb) encoding a single protein, the RNA-dependent RNA polymerase (RDRP) (1–3). *Narnavirus* derives from “naked RNA,” reflecting the absence of a capsid or envelope, and narnaviruses do not form infectious viral particles. Two genera are recognized: *Mitovirus,* found in the mitochondrion of fungi and translated using the mitochondrial genetic code, and cytoplasmic *Narnavirus*, comprising the 20S/23S elements of *Saccharomyces cerevisiae* and one from the oomycete *Phytophthora infestans* (3–5). Here, we report the discovery and complete genome sequence (including termini) of *Phytomonas serpens* narnavirus 1 (PserNV1).

Total cellular RNA from *P. serpens* isolate 9T (6) was extracted using TRIzol reagent (Thermo Fisher), treated with DNase I (Thermo Fisher) at 37°C for 45 min, and purified with RNA Clean & Concentrator-25 (Zymo Research). Replicative viral double-stranded RNAs (dsRNAs) were visualized following treatment with S1 nuclease (Thermo Fisher), separation by agarose gel electrophoresis, and visualization by ethidium bromide staining (7). A prominent dsRNA band of about 4 kb was observed, eluted, and used as a template to generate a cDNA library, with fragment sizes ranging from 200 to 600 nucleotides (nt). A multiplexed TruSeq RNA library was sequenced (2 × 101 cycles, paired-end reads) on the HiSeq 2500 (Illumina). A total of 64,373 reads were obtained, of which 47,690 assembled into a single contig, which was confirmed by reverse transcriptase PCR and sequencing. The adaptor (5′-PO_4_-CCCGTCGTTTGCTGGCTCTTT-NH_2_) was added to the eluted RNA band with T4 RNA ligase (NEB), amplified using primers complementary to the adapter and virus, and sequenced to determine the termini.

The PserNV1 RNA genome is 3,782 nt in length, with a G+C composition comparable to that of the *P. serpens* nuclear genome (48.2 versus 46.6%, respectively) (6). As in other narnaviruses, short hairpins are evident at both the 5′ and 3′ termini. From the first AUG (nt 83), translation with the nuclear genetic code revealed a single open reading frame (ORF) encoding a 1,208-amino-acid protein exhibiting motifs seen in narnaviral RNA-dependent RNA polymerases (1). Phylogenetic comparisons with accepted members of the *Narnaviridae* placed it firmly within the monophyletic genus *Narnavirus*. As the overall RDRP amino acid sequence difference was >80%, this element warrants status as a new *Narnavirus* species.

Members of the genus *Phytomonas* affect >100 plant species, including economically important plants, such as coffee, coconut, cassava, and oil palms (8), which may bear relatives of PserNV1. Classification as a narnavirus suggests that PserNV1 differs from the virus-like particles described previously in other *Phytomonas* species. (9). Some data suggest that PserNV1 may be unstable during culture, as reverse transcriptase PCR tests of this strain acquired independently from another source did not reveal PserNV1 (the authenticity of this strain was confirmed by sequence of two nuclear genes, *GAPDH* and *PTR1*). In the future, studies of NV1-positive and -negative lines of *Phytomonas* may establish potential roles of this viral element in plant pathogenicity (1).

### Nucleotide sequence accession number.

The full-length viral genomic sequence of PserNV1 from *Phytomonas serpens* 9T strain was deposited in GenBank under the accession no. KU882057.
